# Study on lightening of coal tar with metal oxide supported γ-Al_2_O_3_ catalyst

**DOI:** 10.1038/s41598-023-45589-z

**Published:** 2023-10-26

**Authors:** Chen Jihao, Li Juan, Li Yue, Zhang Jinjing

**Affiliations:** 1 Shaanxi Coalfield Geological Engineering Technology Co., Ltd, Xi’an, 710054 China; 2 Key Laboratory of Coal Resources Exploration and Comprehensive Utilization, Ministry of Natural Resources, Xi’an, 710021 China

**Keywords:** Environmental sciences, Energy science and technology

## Abstract

In this experiment, a fixed bed of pyrolysis was used to conduct pyrolysis with coal and a mixture of coal and catalyst, and the distribution and composition of tar products were studied. The pyrolysis of raw coal was carried out at different temperatures and at different constant temperature times, and the effects of pyrolysis temperature and constant temperature pyrolysis time on tar product formation from raw coal pyrolysis were studied. γ-Al_2_O_3_ was used as the carrier, and 4 kinds of alkaline earth metal oxides (MgO, CaO, SrO, BaO), 3 kinds of subgroup metal oxides (Fe_2_O_3_, Co_2_O_3_, NiO) and 5 kinds of VIII metal oxides (Cr_2_O_3_, MnO_2_, CuO, ZnO, MnO_2_) were selected as active components. The supported γ-Al_2_O_3_ catalyst was prepared by the method of equal volume impregnation and roasting in a muffle furnace. The γ-Al_2_O_3_ catalyst was characterized by means of XPS, BET and SEM, and the mechanism of the mixed pyrolysis of coal with different metal oxide supported catalysts to generate tar was studied. The results showed that: (1) under the conditions of 450 °C, 500 °C, 550 °C and 600 °C, the maximum tar yield was 0.32 g at 600 °C, and the tar yield was higher at constant temperature for 15 min than at final temperature of 600 °C, with an increase of 15.63%. (2) Fe_2_O_3_/γ-Al_2_O_3_ catalyst resulted in the highest tar yield of 0.75 g, which was 134.38% higher than that of coal pyrolysis. (3) From the increase of light oil and phenol oil and the decrease of anthracene oil and asphalt, Co_2_O_3_/γ-Al_2_O_3_, Fe_2_O_3_/γ-Al_2_O_3_ and Cr_2_O_3_/γ-Al_2_O_3_ can improve the tar quality better.

## Introduction

With the development of science and technology, there are many new energy sources, such as solar energy, geothermal energy, wind energy and so on, and the use of these new energy sources accounts for a certain proportion of the total energy consumption^1^, but the position of coal and oil in energy utilization is still irreplaceable. China's energy structure is characterized by “rich coal, less oil, poor gas”^2^, which determines that China will continue to use coal as a major energy for a considerable period of time. The direct combustion of coal produces a large amount of pollutants, causing great damage to the environment, such as the greenhouse effect, ozone destruction, acid rain, etc. At the same time, the direct combustion of coal will also cause a lot of waste^3^, reducing the use value of coal. China's coal resources contain a large amount of low-rank coal. The clean utilization of low-rank coal and the realization of low-rank coal polygeneration have always been the research hotspot of many experts and scholars^4^, and great progress has been made, for example, the production of water gas and coal liquefaction have achieved industrial production. There are still many problems in the process of pyrolysis of low rank coal, so it is very important to reduce environmental pollution and ease the pressure of oil import by pyrolysis of low rank coal.

Coal pyrolysis is the most basic and critical way to realize coal-to-gas and coal-to-oil. With the increase of pyrolysis temperature, coal undergoes a series of changes, eventually generating pyrolysis coke, gas and coal tar. Coal tar contains a lot of chemical substances, and the light coal tar produced by pyrolysis can be used to extract high value-added chemicals, gasoline, diesel, etc.^5^ which can make use of the precious hydrocarbon resources in coal. However, the tar produced by the direct pyrolysis of coal is easy to block the pipeline and corrode equipment, and the heavy tar content in the tar is high, and the utilization rate of the direct pyrolysis product is also very low^6^. How to improve the yield and quality efficiency of coal pyrolysis products is a hot research topic of many scholars. The methods of improving coal pyrolysis products include hydrogenation catalysis and cracking catalysis. In the process of coal pyrolysis, the yield and quality of products are improved by adding catalysts, and the preparation of catalysts with high catalytic effect is a key step to improve the yield and quality of coal pyrolysis products.

There are many studies on the preparation of supported catalysts using metal oxides as coal pyrolysis cracking catalysts. CoO is loaded on coal semi-coke to prepare a catalyst for tar cracking to crack coal pyrolysis products, which increases the mass yield and content of light components in tar by 8.8% and 28.8%, respectively^7^. Liu cracked Pingshuo coal rapid pyrolysis tar after loading Mo into HZSM-5, which greatly increased the amount of benzene, toluene, ethylbenzene, xylene and naphthalene in the tar product^8^. Liao^9^ selected four metal oxides (CaO, Al_2_O_3_, Fe_2_O_3_, NiO) as catalysts for pyrolysis of Huolinhe lignite. The catalysts have different effects on different pyrolysis stages of coal, and they can promote the conversion rate of lignite pyrolysis. The order of action is: NiO > CaO > Fe_2_O_3_ > Al_2_O_3_; the catalytic effect on lignite in the active pyrolysis stage is: NiO > Fe_2_O_3_ > CaO > Al_2_O_3_, and the catalytic effect on lignite in the thermal polycondensation stage is: Fe_2_O_3_ > CaO > NiO > Al_2_O_3_. Wang^10^ used Co-Mo/Al_2_O_3_ and Ni-Mo/Al_2_O_3_ to catalyze coal pyrolysis, which has a significant effect on improving the lightening of pyrolysis tar, and can increase the yield of products such as light aromatics and naphthalenes in tar, so that the relative mass fraction of light aromatics reaches 40.94% to 50.89%. Cui^11^ used MgO, Fe_2_O_3_, Co_2_O_3_, ZnO, NiO oxide-supported Al_2_O_3_ catalysts to influence the pyrolysis gas products of Huangling coal, and Al_2_O_3_ was used as a carrier to have good mechanical properties and catalytic performance. It can be seen from the above studies that metal oxides have good effects on coal pyrolysis and cracking, and can be used as active components of supported catalysts. Al_2_O_3_ has become an excellent catalyst carrier due to its good physical and mechanical properties. At present, there were many researches on coal pyrolysis to prepare tar at home and abroad. Most of the tar was prepared and modified by pyrolysis/catalysis. It was also common to study the utilization of supported metal oxide catalysts for coal pyrolysis to prepare tar. However, the research on coal pyrolysis to tar was lack of metal oxide supported catalysts of different groups. Therefore, a series of metal oxide-supported catalysts for coal pyrolysis to prepare tar were proposed in this study, which is an innovative study.

Therefore, in this study, a fixed-bed pyrolysis reactor was used to prepare metal oxide-supported γ-Al_2_O_3_ catalysts by muffle furnace calcination. The catalyst and coal are mixed and pyrolyzed to control the tar and improve the quality. It will provide a theoretical and practical basis for the comprehensive utilization of coal pyrolysis product resources, and further develop coal clean technology.

## Experiment

### Experimental materials

The coal sample used in this paper is produced in Cuimu mining area of Shaanxi Province. The coal sample is broken up to 3 mm in particle size. The catalyst used in the experiment was a spherical γ-Al_2_O_3_ with a particle size of 3 mm purchased on the market.

### Experimental drugs

Fe(NO_3_)_3_·9H_2_O, Co(NO_3_)_2_·6H_2_O, Ni(NO_3_)_2_·6H_2_O, Cu(NO_3_)_2_·3H_2_O, Cr(NO_3_)_3_·9H_2_O and Mn(NO_3_)_2_·4H_2_O are all analytical pure reagents produced by Chemical Reagent Co., Ltd. of National Pharmaceutical Group.

MgCl_2_·6H_2_O, CaCl_2_, SrCl_2_·6H_2_O, BaCl_2_·2H_2_O, (CH_2_COO)_2_Zn·2H_2_O and (NH_4_)_6_Mo_7_O_24_·4H_2_O are analytical pure reagents produced by Tianjin Beilian Fine Chemicals Development Co., Ltd.

### Preparation of catalyst

Preparation of alkaline earth metal oxide catalyst supported on γ-Al_2_O_3_: The equal volume impregnation method was used^12–14^. 3g γ-Al_2_O_3_ was immersed in 5% MgCl_2_ solution for 24 h and then removed and placed in a muffle oven at 500 °C for 4 h. The MgO/γ-Al_2_O_3_ catalyst with 5% loading was removed after cooling to room temperature. CaO/γ-Al_2_O_3_, SrO/γ-Al_2_O_3_ and BaO/γ-Al_2_O_3_ catalysts were prepared by the same method.

Preparation of subgroup metal oxide catalyst supported on γ-Al_2_O_3_: the equal volume impregnation method was used. 3 g γ-Al_2_O_3_ was immersed in 5% Fe(NO_3_)_3_ solution for 24 h and then removed and placed in a muffle oven at 500 °C for 4 h. The Fe_2_O_3_/γ-Al_2_O_3_ catalyst with 5% loading was removed after cooling to room temperature. Co_2_O_3_/γ-Al_2_O_3_ and NiO/γ-Al_2_O_3_ catalysts were prepared by the same method.

Preparation of VIII metal oxides catalyst supported on γ-Al_2_O_3_: The equal volume impregnation method was used. 3 g γ-Al_2_O_3_ was immersed in 5% (CH_2_COO)_2_Zn solution for 24 h and then removed and placed in a muffle oven at 500 °C for 4 h. The ZnO/γ-Al_2_O_3_ catalyst with 5% loading was removed after cooling to room temperature. Mo_2_O_3_/γ-Al_2_O_3_, Cr_2_O_3_/γ-Al_2_O_3_ and MnO_2_/γ-Al_2_O_3_ catalysts were prepared by the same method.

### Experimental evaluation

**Figure 1:**
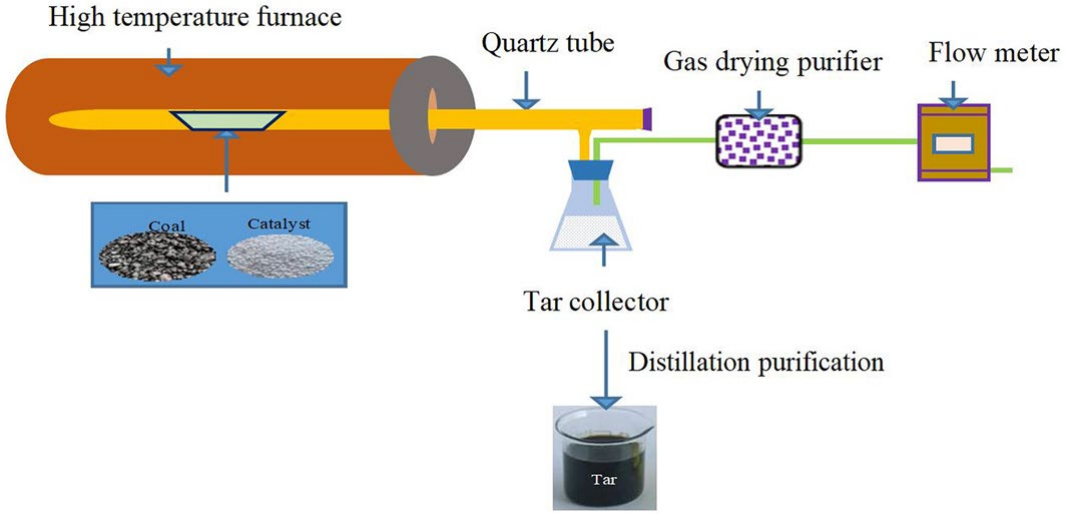
Coal Tar catalytic cracking process flow diagram.

The catalytic cracking process of coal tar is shown in Fig. 1.

The 10 g coal sample and the 3 g catalyst were mixed evenly and placed in the pyrolysis furnace. During the experiment, the temperature of catalyst and coal were from 24 to 600 °C, and the temperature was constant at 600 °C for a period of time. With the increase of temperature, the gaseous producted from coal pyrolysis to the tar collection bottle, and the tar components and the tar produced was analyzed by GC.

### Tar detection

The detection of coal tar is mainly carried out by means of simulated distillation to analyze the distribution of each fraction in the tar, and is carried out on a simulated distillation chromatogram. The principle of this method is a non-polar column with a certain degree of separation, testing the retention time of known mixture components under linear temperature programming conditions. Then, under the same chromatographic conditions, the samples are sequentially separated according to the boiling points of the components, and the slice integration is performed to obtain the corresponding cumulative area, and the corresponding retention time. After temperature–time interpolation correction, a temperature corresponding to a percent yield, that is, a distillation range, is obtained, wherein the cumulative area percentage is the yield. Table1 shows the fractions and corresponding boiling points of simulated distillation gas chromatography plus coal tar measurement. The simulated distillation experiment was used to determine the tar components in this experiment. The dewatered tar samples were filtered in acetone solvent, and the prepared tar samples were prepared. Set operating conditions, injection mode: on-column, gasification temperature: 350 °C, column temperature: initial temperature: 50 °C, initial time: 0 min, heating rate: 9 °C/min, final temperature: 360 °C, final time: 1.5 min, column flow rate (high purity N_2_): 5 mL/min, gas (high purity H_2_): 30 mL/min, combustion-supporting gas (purified Air): 360 mL/min, supplementary gas (high purity N_2_): 25 mL/min, Sample Volume: 0.2–0.5 mL, operation time: 36 min.

**Table 1:** Boiling points range for classifying tar fractions.

Coal tar fraction	Light oil	Phenolic oil	Naphthalene oil	Washing oil	Anthracene oil	Asphalt
Boiling point (℃)	< 170	170–210	210–230	230–300	300–360	> 360

### Characterization of the catalyst

Thermogravimetric analysis of coal samples: the raw coal is a Swiss Mettler-Toledo TGA/SDTA851e thermogravimetric analyzer. During the experiment, the carrier gas was selected from high purity N_2_, the gas flow rate was 60 mL/min, the temperature range was 24–900 °C, and the heating rate was 10 °C/min.

The Vario EL III element analyzer (Elementar company in Germany) was used for elemental detection of coal samples.

Specific surface area analysis (BET): The ASP 2460 type surface and pore size analyzer is used to measure the specific surface area of different types of catalysts.

Scanning electron microscope (SEM): JSM-6460LV, working voltage 20 kV, magnification 5000 times.

X-ray electron spectroscopy (XPS): XPS was an important tool for analyzing the surface structure and composition of an element, and it can get the composition of the test object.

### Coal sample analysis

#### Industrial and elemental analysis of coal samples

Table 2 is the industrial analysis and elemental analysis of coal samples. The ash content of coal sample industrial analysis is determined according to the national standard GB/T212-2008. The coal sample is burned to a constant quality at a temperature of (815 ± 10) ℃, the burning atmosphere is air, and the combustible organic components in the coal are burned; Thermogravimetric analysis is to analyze and determine in the nitrogen atmosphere, and some organic components in coal will not be decomposed; There is a huge difference between the two test conditions, and the ash obtained by the test is very different. The third transition/peak (about 760 °C) in the DTG curve is caused by the pyrolysis and precipitation of asphaltic substances (difficult to decompose macromolecular organic components) in coal, which represents the precipitation temperature of asphaltic substances.

**Table 2:** Industrial analysis and the elemental analysis of lignite coal (%).

Industrial analysis				Elemental analysis				
M_ad_	A_ad_	V_ad_	FC	C	H	O	N	S
4.99	11.87	32.79	50.35	67.64	3.63	27.53	0.67	0.53

#### Analysis of coal samples

**Figure 2:**
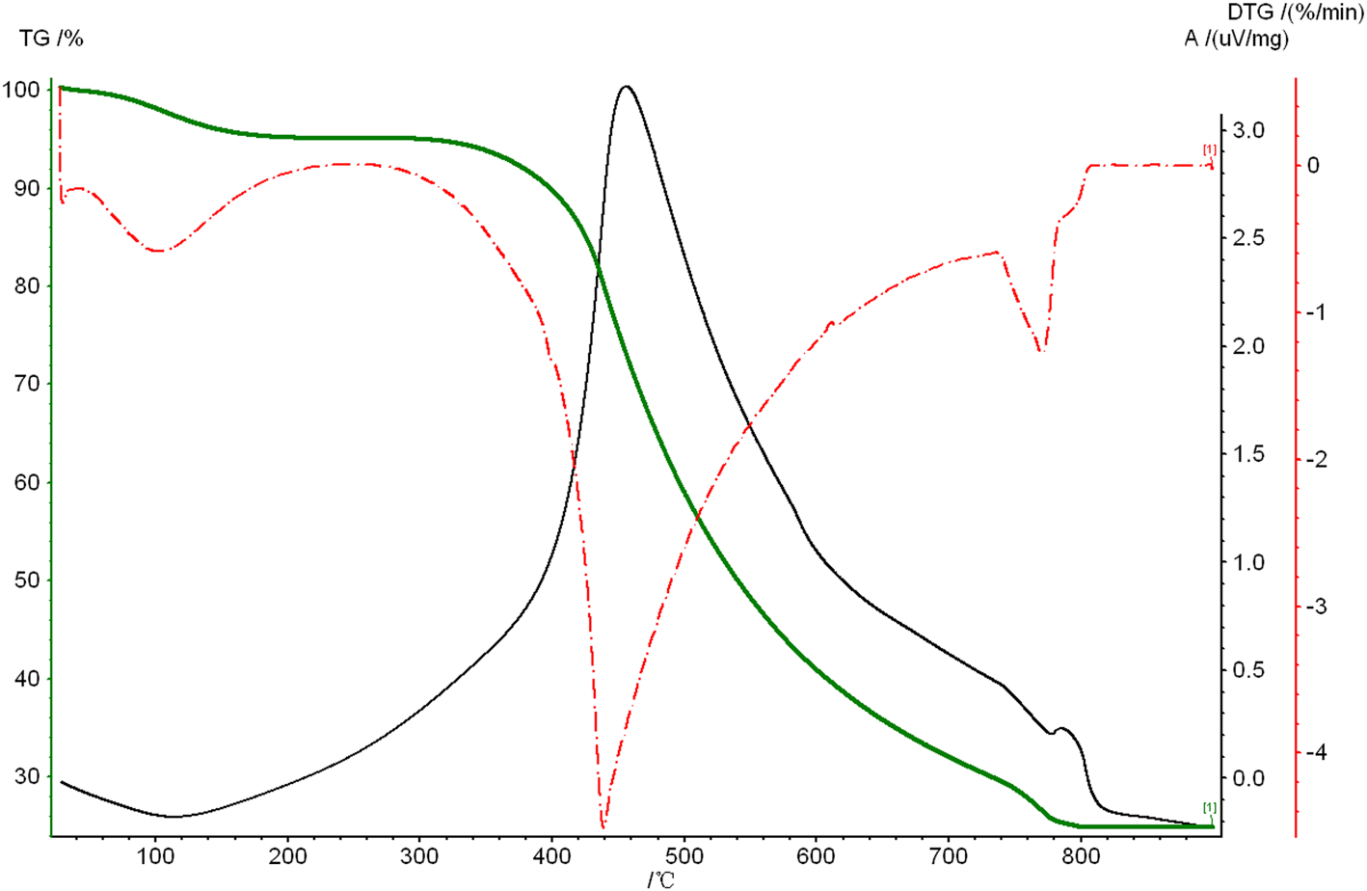
TG and DTG curves of coal.

N_2_ atmosphere, heating rate 10 °C/min, weight loss curve (TG) and weight loss rate curve (DTG) are shown in Fig. 2.

It can be seen from Fig. 2 that the weight loss of coal samples is divided into four stages. The first stage is between 24 and 150 °C. This stage is the drying stage, which is mainly the precipitation of moisture and adsorbed gas. The second stage is between 150 and 300 °C, this stage is the preheating stage, no obvious pyrolysis phenomenon occurs, and the TG and DTG curves have no obvious changes. The third stage is between 300 and 700 °C, which is the pyrolysis stage of coal, which is accompanied by the decomposition of functional groups such as phenolic carboxyl groups with poor thermal stability in the molecular structure, and the bridges between aromatic rings in the macromolecular network structure. The breakage of bonds and aliphatic side chains releases a large amount of gaseous hydrocarbons and tar vapors, and the coal sample loses weight rapidly and reaches the maximum weight loss rate. The DT curve of the coal sample drops sharply after 400 °C, and the DTG curve also shows the highest peak of weight loss. The peak temperature of pyrolysis is 450 °C, which is the temperature corresponding to the maximum weight loss temperature. The maximum weight loss temperature reflects the stability of the macromolecular structure of the coal. The lower the peak temperature, the more easily the network structure in the coal is destroyed, and the higher the reactivity of the coal is, the more unstable the structure is during the pyrolysis process^15,16^.

## Results and discussion

### Effects of pyrolysis temperature and constant temperature pyrolysis time on the distribution of tar products

#### Effect of pyrolysis temperature on tar yield and tar composition

**Figure 3:**
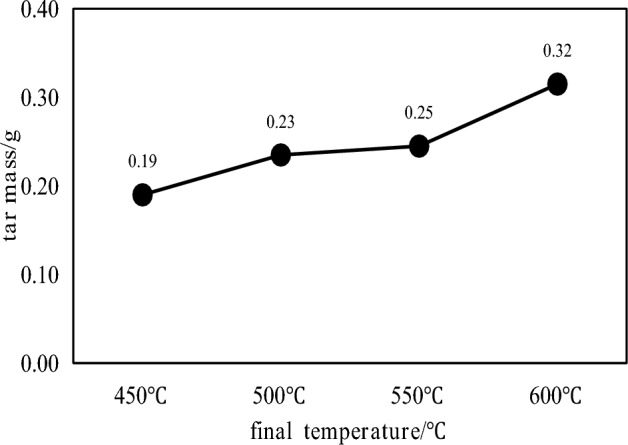
Distribution of tar yield at different pyrolysis temperatures.

**Figure 4:**
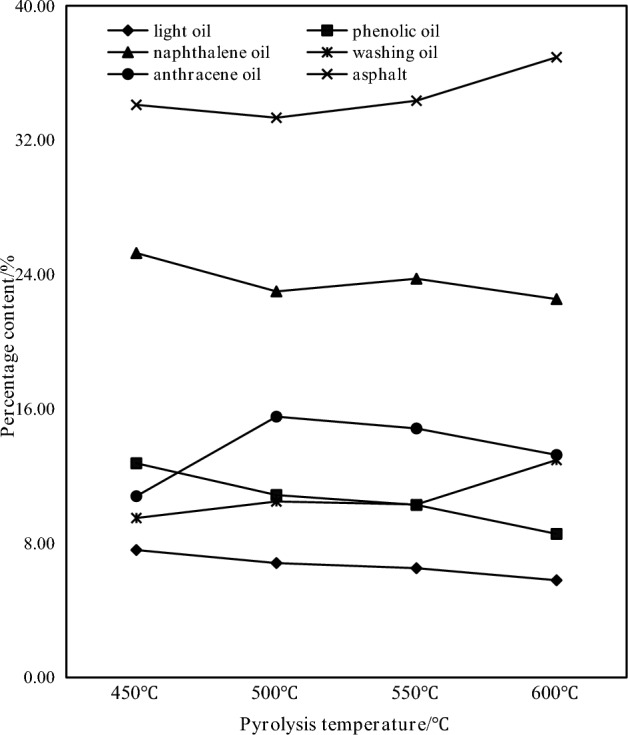
Distribution of tar components at different pyrolysis temperatures.

The coal tar yield and tar component distribution at different pyrolysis temperatures are shown in Figs. 3 and 4, respectively.

It can be seen from Fig. 3 that the tar yield increases with the increase of the pyrolysis temperature.When the pyrolysis temperature is 450 °C, the tar yield is 0.19 g, and when the pyrolysis temperature is 600 °C, the tar yield is 0.32 g, which is 68.4% higher than that at 450 °C. The tar produced by the pyrolysis and polycondensation of coal at 600 °C basically reaches the maximum value. It can be seen from Fig. 4 that with the increase of the pyrolysis temperature, the proportion of most of the light fractions in the collected coal tar showed a decreasing trend, and the proportion of washing oil and asphalt showed an increasing trend. It shows that the higher the pyrolysis temperature, the less the total content of light fractions in the collected coal tar and the more pitch. The specific content of each fraction of coal tar also changs at different pyrolysis temperature. The content of light oil, phenol oil and naphthalene oil decreases with the increase of pyrolysis temperature, and the content of anthracene oil first increases and then decreases with the increase of pyrolysis temperature. The pyrolysis temperature has an effect on the degree of coal cracking reaction. When the temperature is low, light components are produced and surface overflow in the coal. With the increase of temperature within 600 °C, the heavier components with larger molecular weights are cracked, the more asphalt products are cracked, and the overall content of light fractions in the collected coal tar decreases.

#### Influence of constant temperature time on tar composition

**Figure 5:**
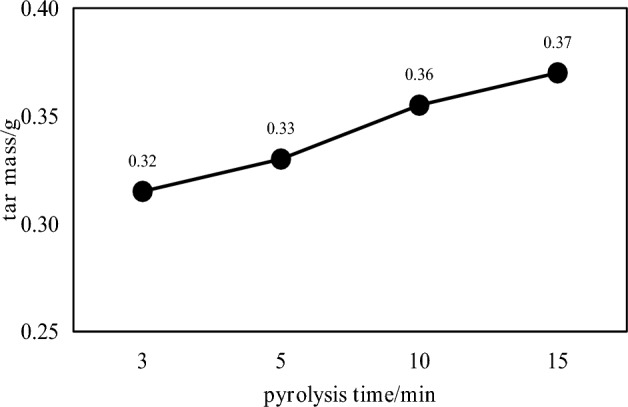
Distribution of coal tar production under different constant temperature pyrolysis times.

**Figure 6:**
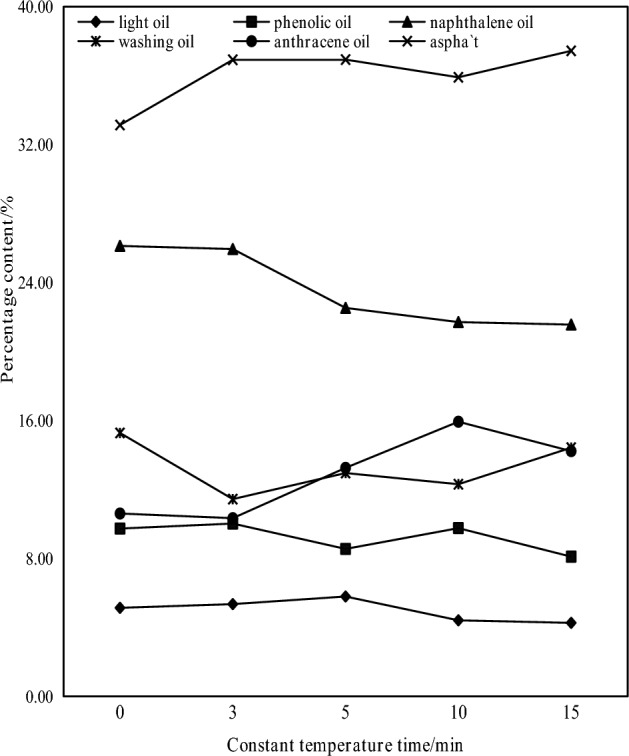
Distribution of tar components under different constant temperature pyrolysis time.

The coal tar yield and tar component distribution under different constant temperature pyrolysis times are shown in Figs. 5 and 6, respectively.

It can be seen from Fig. 5 that when the final temperature of pyrolysis is 600 °C, the tar yield also increases slowly with the increase of constant temperature time. The tar yield of constant temperature pyrolysis for 3 min was 0.32 g, while that of constant temperature pyrolysis for 15 min was 0.37 g. The tar yield of constant temperature pyrolysis for 15 min increased by 15.63% compared with the final pyrolysis temperature of 600 °C. However, the output of pyrolysis gas has been increasing. Since the final temperature of pyrolysis is 600 °C, the secondary degassing is mainly polycondensation, and the volatile components are mainly hydrocarbon gases, hydrogen and carbon oxides, and less tar is produced. It can be seen from Fig. 6 that with the increase of constant temperature pyrolysis time, the proportion of most of the light fractions in the collected coal tar showed a decreasing trend, and the proportion of washing oil and asphalt showed an increasing trend. It shows that the total content of light fractions in coal tar decreases with the increase of constant temperature time, and the asphalt increases. With the increase of constant temperature pyrolysis time, the content of light oil, phenol oil and naphthalene oil decreases. With the increase of constant temperature pyrolysis time, the content of anthracene oil first increases and then decreases. The main reason is that the constant temperature pyrolysis time is short, and the tar macromolecules produced cannot be quickly overflowed in the coal. With the prolongation of the constant temperature pyrolysis time, the tar macromolecular substances overflow in the coal, which increases the heavy tar content in the tar components. The light component content is also reduced accordingly.

### Effects of different groups of metal oxide-supported catalysts mixed with coal on the distribution of tar products in pyrolysis

#### Effect of alkaline earth metal oxide supported catalyst on tar components

**Figure 7:**
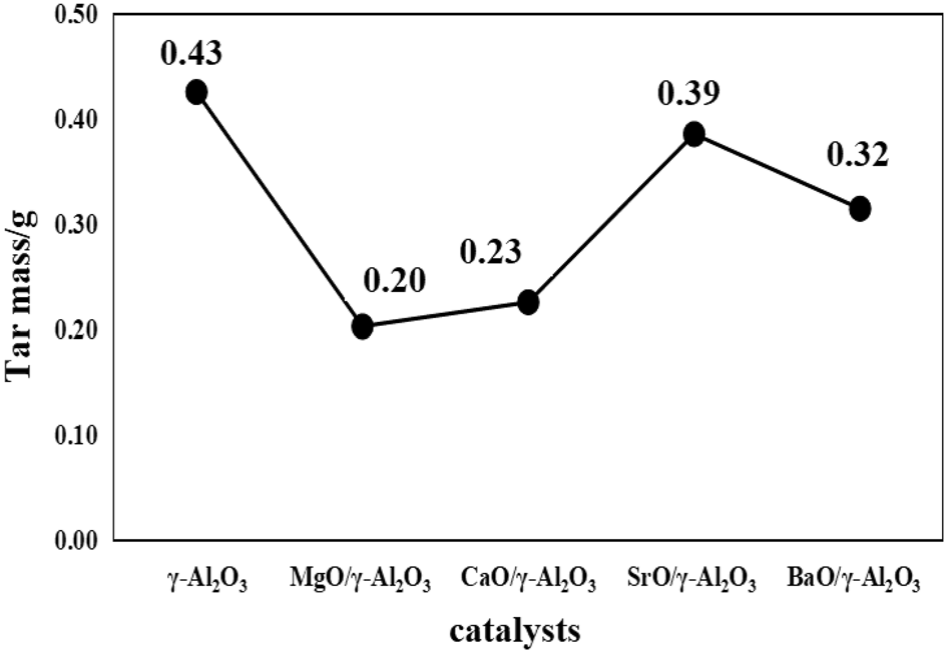
Distribution of coal tar production by alkaline earth metal oxide supported catalysts.

**Figure 8:**
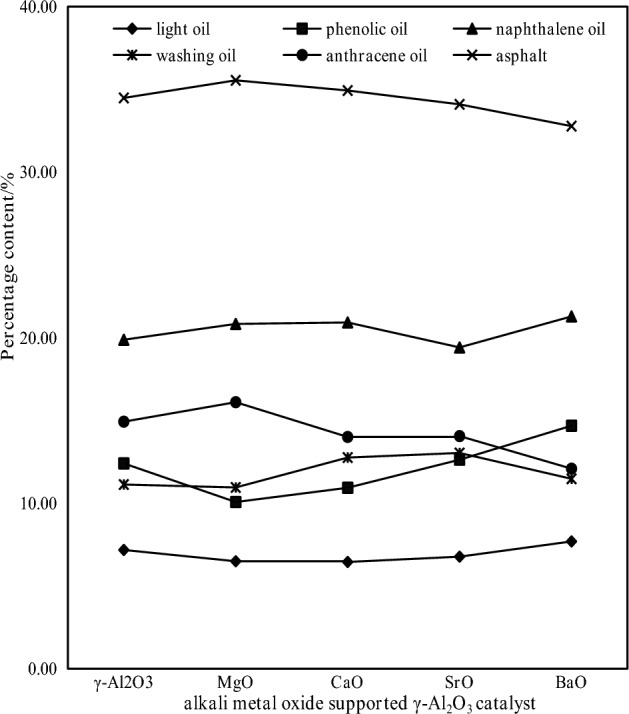
Distribution of tar components by alkaline earth metal oxide supported catalysts.

The coal tar yield and tar composition distribution of the alkaline earth metal oxide supported catalyst are shown in Figs. 7 and 8, respectively.

Figure 7 shows that the addition of alkaline earth metal oxide-supported γ-Al_2_O_3_ catalyst can catalyze the pyrolysis of coal and increase tar production^17^. After adding γ-Al_2_O_3_ and SrO/γ-Al_2_O_3_, the tar yield increased by 27.27% and 18.18% compared with that of 600 °C, respectively. Therefore, it can be concluded that γ-Al_2_O_3_ and SrO/γ-Al_2_O_3_ have a significant effect on the tar yield. From Fig. 8, it can be seen that the content of light tar increases and the content of asphalt decreases after adding alkaline earth metal catalyst, which shows that the supported catalyst is beneficial to the production of light tar. The study found that the carrier γ-Al_2_O_3_ tar cracking played a catalytic role, increasing the proportion of light oil and phenol oil in the tar. Compared with the pyrolysis of raw coal, the percentage of light oil and phenol oil increases by 23.99% and 45.14%, respectively. The BaO active component tar has the best cracking effect, and its light oil and phenol oil percentages increases the most, increasing by 32.88% and 71.75%, respectively, and the asphalt percentage decreases the most, decreasing by 11.24%. Through the specific surface area test, γ-Al_2_O_3_ has a large specific surface area, which is conducive to the dispersion of active metal oxides, and can form smaller crystal grains. surface area, which is favorable for catalytic reactions.

#### Influence of subgroup metal oxide supported catalyst on tar components

**Figure 9:**
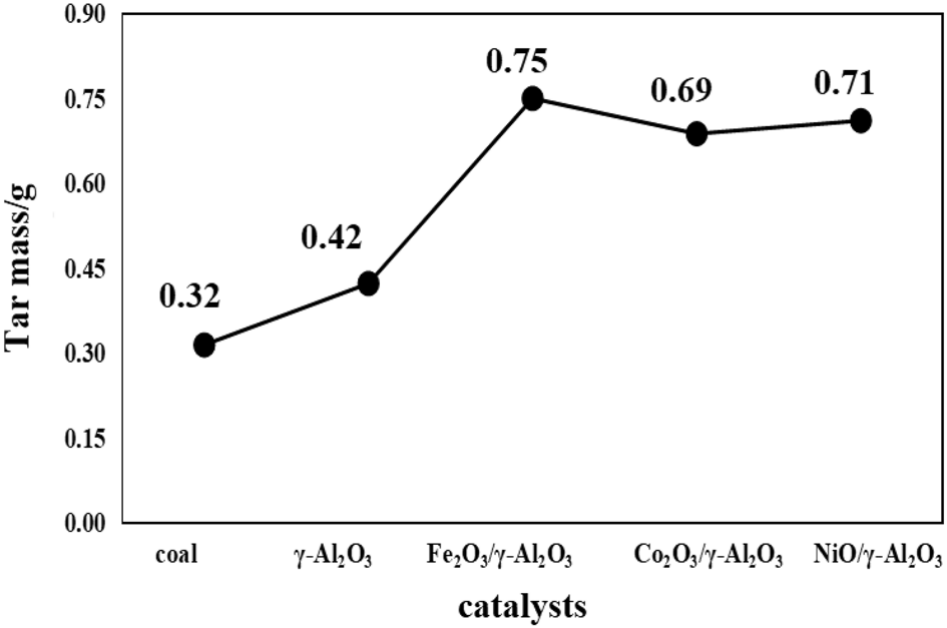
Distribution of coal tar production by subgroup metal oxide supported catalysts.

**Figure 10:**
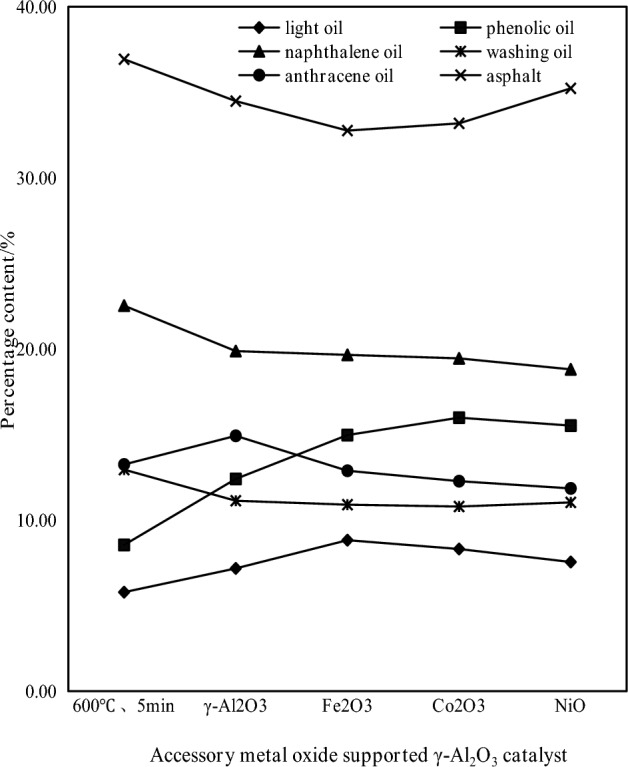
Distribution of coal tar components by subgroup metal oxide supported catalysts.

The coal tar yield and tar composition distribution of the subgroup metal oxide-supported catalyst are shown in Figs. 9 and 10, respectively.

It can be seen from Fig. 9 that the tar yield increases after adding γ-Al_2_O_3_, Fe_2_O_3_/γ-Al_2_O_3_, CoO/γ-Al_2_O_3_ and NiO/γ-Al_2_O_3_ during the coal pyrolysis process; Fe_2_O_3_/γ-Al_2_O_3_ makes the liquid yield the highest, which is 0.75 g, 134.38% higher than that of raw coal. However, after the active materials Fe_2_O_3_, CoO and NiO are loaded on γ-Al_2_O_3_, the active sites are increased on the surface and inside of γ-Al_2_O_3_, which further promotes the cracking of macromolecular substances, thereby further increasing tar, which Fe_2_O_3_ makes the activity of the catalyst the highest. It can be seen from Fig. 10 that the composition of the tar produces by adding the subgroup metal oxide catalyst changes differently. The content of light oil and phenol oil in the tar increases significantly, while the content of naphthalene oil, washing oil, anthracene oil and asphalt decreases. Fe_2_O_3_/γ-Al_2_O_3_, Co_2_O_3_/γ-Al_2_O_3_, NiO/γ-Al_2_O_3_ compared with γ-Al_2_O_3_ mixed pyrolysis found that light oil and phenol oil increased by 22.98% and 20.67%, 15.79% and 28.85%, 5.22% and 25.12%, respectively; the cracking effect of anthracene oil was obvious, which decreased by 13.68%, 17.76% and 20.60% respectively. Fe_2_O_3_/γ-Al_2_O_3_ crackes the most asphalt, and the asphalt content decreases by 4.99%. From the increase of light oil and phenol oil and the decrease of anthracene oil and asphalt, Co_2_O_3_/γ-Al_2_O_3_ and Fe_2_O_3_/γ-Al_2_O_3_ have better effect on coal pyrolysis.

#### Effect of group VIII metal oxide supported catalysts on tar composition

**Figure 11:**
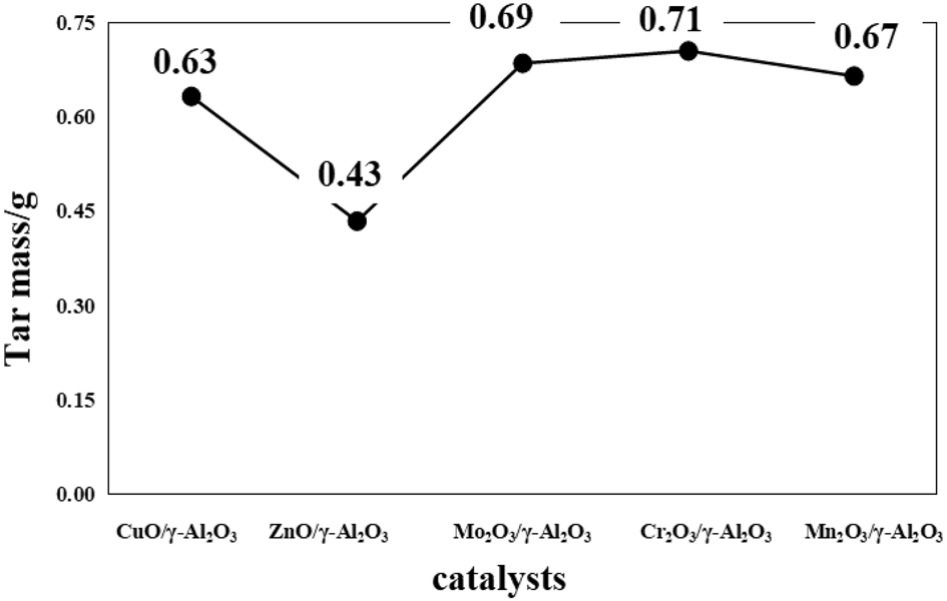
Distribution of coal tar production by Group VIII metal oxide supported catalysts.

**Figure 12:**
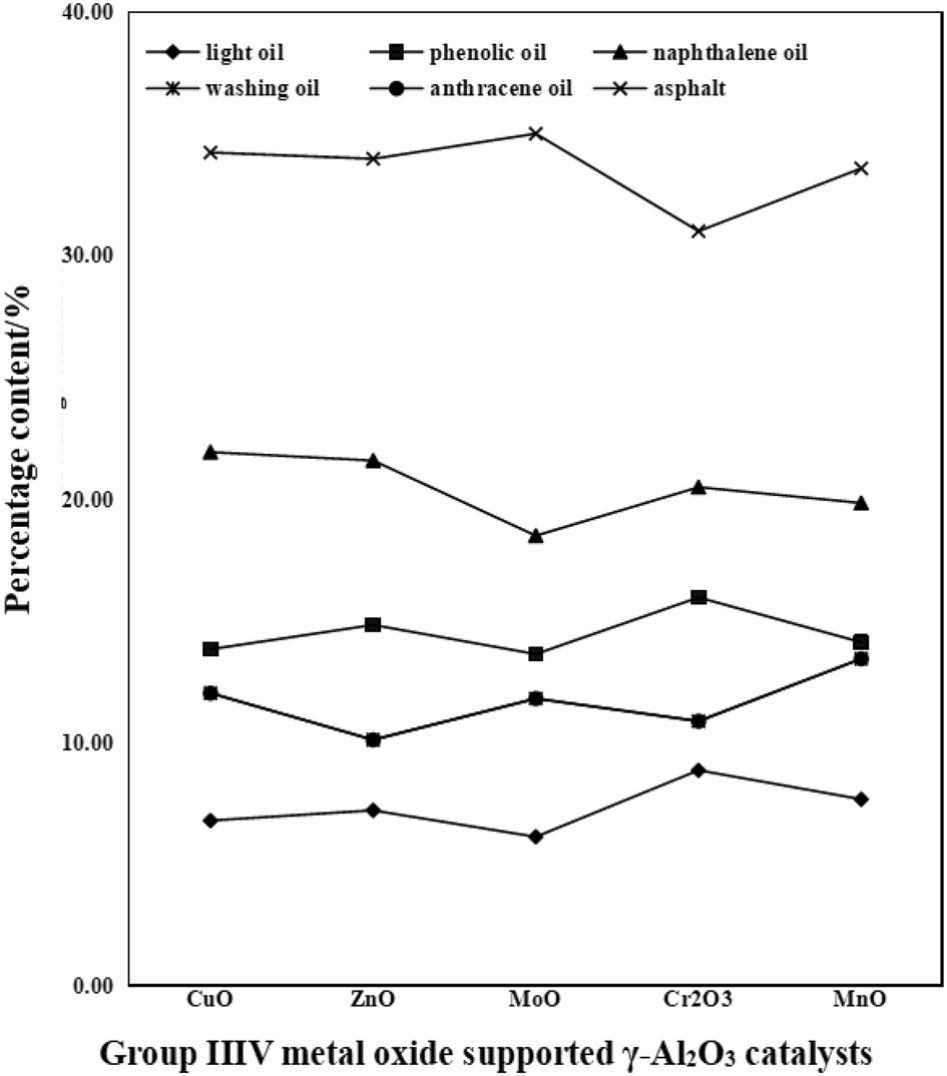
Distribution of coal tar components by Group VIII metal oxide supported catalysts.

The coal tar yield and tar composition distribution of the Group VIII metal oxide supported catalysts are shown in Figs. 11 and 12, respectively.

It can be seen from Fig. 11 that the tar yield is improved after adding Group VIII metal oxide catalyst in the coal pyrolysis process; Cr_2_O_3_/γ-Al_2_O_3_ makes the tar yield the highest at 0.71 g, which is 121.82% higher than that of raw coal. It can be seen from Fig. 12 that after adding the Group VIII metal oxide supported catalyst, the content of phenol oil and washing oil in the tar increases significantly, while the content of anthracene oil and pitch decreases. Compared with γ-Al_2_O_3_ mixed pyrolysis of Cr_2_O_3_/γ-Al_2_O_3_, it is found that light oil and phenol oil changed by 23.45% and 28.67% respectively; the cracking effect of anthracene oil is obvious, which decreases by 27.12%; Cr_2_O_3_/γ-Al_2_O_3_ crackes the most asphalt, and asphalt content decreases by 10.09%. From the increase of light oil and phenol oil and the decrease of anthracene oil and bitumen, Cr_2_O_3_/γ-Al_2_O_3_ has the best effect on coal pyrolysis. When the coal sample is mixed with Cr_2_O_3_/γ-Al_2_O_3_ for pyrolysis, the coal sample is in contact with the active sites on the surface of Cr_2_O_3_/γ-Al_2_O_3_, which makes the macromolecular chain in the coal break, which promotes the pyrolytic cracking of the coal and generates more heat. solution product. The generated pyrolysis product enters into the interior of Cr_2_O_3_/γ-Al_2_O_3_ and contacts with active Cr_2_O_3_, which further breaks the macromolecules to produce small molecular substances, which are easily vaporized and volatilized at higher temperatures. Therefore, Cr_2_O_3_/γ-Al_2_O_3_ results in the highest tar yield and the best catalytic effect during the mixed pyrolysis process.

## Catalyst characterization analysis

### SEM characterization

**Figure 13:**
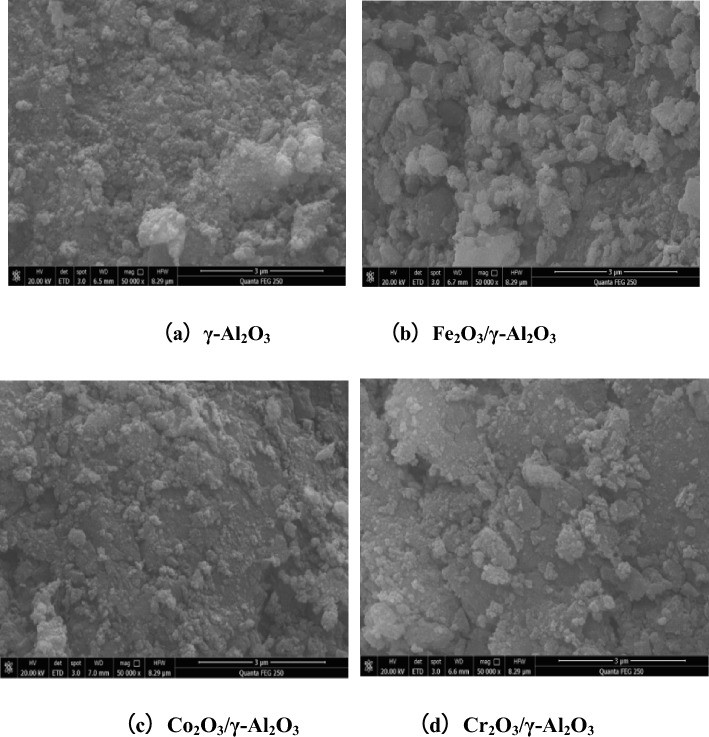
SEM image of supported γ-Al_2_O_3_ catalyst.

The SEM characterization results of catalysts loaded with different metal oxides are shown in Fig. 13.

It can be seen from Fig. 13 that the surface of γ-Al_2_O_3_ is uneven, the particle distribution on the surface is uneven, and there is no fixed form. But it can be seen that the surface of γ-Al_2_O_3_ has a relatively rich pore structure, which is conducive to the dispersion of active components. The interior presents an irregular mass structure, and the inner space of the pores is large^18^. The surface morphologies of catalysts loaded with different metal oxides changes, compared with γ-Al_2_O_3_. It can be seen that the Fe_2_O_3_/γ-Al_2_O_3_ surface is distributed with a flaky structure with non-uniform size and irregular shape. At the same time, it is found that there are slight cracks on the surface of the catalyst, which may be caused by the rapid heating rate during the calcination process. The surface of Co_2_O_3_/γ-Al_2_O_3_ and Cr_2_O_3_/γ-Al_2_O_3_ is unevenly dispersed with a large number of particles, and there is agglomeration structure, and the surface particles of Cr_2_O_3_/γ-Al_2_O_3_ are not relatively large.

### XPS characterization

**Figure 14:**
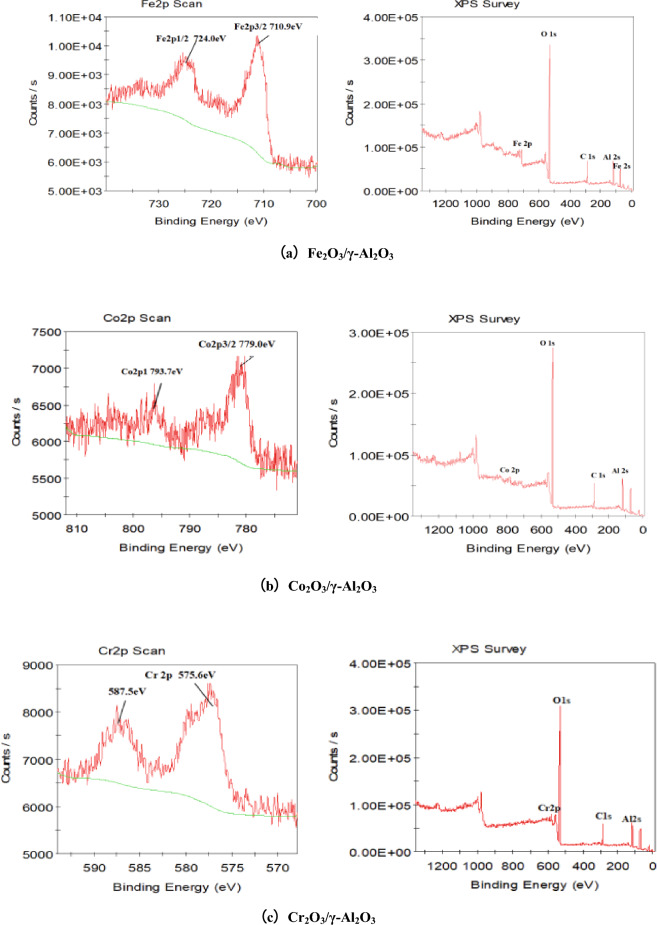
XPS of supported γ-Al_2_O_3_ catalyst.

The chemical composition of Fe_2_O_3_/γ-Al_2_O_3_, Co_2_O_3_/γ-Al_2_O_3_ and Cr_2_O_3_/γ-Al_2_O_3_ catalysts was analyzed by XPS in Fig. 14a–c, respectively. Figure 14a shows the decomposition pattern of active metal Fe and the scanning diagram of the catalyst. Fe, O and Al are mainly present on the surface of Fe_2_O_3_/γ-Al_2_O_3_ catalyst, which is consistent with the preparation process of the catalyst, the Electron binding energy of Fe2p3/2 is at 710.9 eV, and that of Fe2p1/2 is at 724.0 eV. The peak of Fe2p corresponds to that of Fe_2_O_3_, which indicates that the active component Fe exists in the form of Fe_2_O_3_ in Fe_2_O_3_/γ-Al_2_O_3_ catalyst^19^. Figure 14b shows the decomposition pattern of active metal element Co and the full scan picture of catalyst. Co, O and Al are mainly present on the surface of Co_2_O_3_/γ-Al_2_O_3_ catalyst, which is consistent with the preparation process of catalyst. The binding energies of Co^3+^ and Co^2+^ XPS peaks are 780.3 eV and 781.9 eV respectively, and the satellite peak located at 793.7 eV corresponds to Co^2+^ species^12^. Co_2_O_3_ and CoO exist in the form of active components in Cr_2_O_3_/γ-Al_2_O_3_ catalyst. Figure 14c shows the decomposition pattern of the active metal Cr and the scanning diagram of the catalyst. There are Cr, O and Al on the surface of Cr_2_O_3_/γ-Al_2_O_3_ catalyst, which is consistent with the preparation process of the catalyst, the decomposition map of the active metal Cr, with strong Cr2p characteristic peaks around 575.6 ev and 587.5 eV, indicates the presence of Cr_2_O_3_^20^. The C1s peak also appeared in the XPS full spectrum of the three catalysts, which was caused by the contaminated carbon adsorbed on the surface of the samples in the laboratory environment.

### BET characterization

Table 3 shows the specific surface area of different metal oxide catalysts. It can be seen from Table 3 that the specific surface area of the carrier γ-Al_2_O_3_ is 271.64 m^2^/g. The specific surface area of the catalyst supported by the subgroup metal oxide changed. The supported catalyst will form oxide particles inside, thereby increasing the specific surface area of the catalyst. The specific surface area of Ni_2_O_3_/γ-Al_2_O_3_ increases and decreases to 250.20 m^2^/g. The reason for the decrease of the specific surface area of the catalyst loaded with Ni_2_O_3_ may be due to the continuous deposition of active metal particles on the surface of the carrier, resulting in the reduction of catalyst pores or partial clogging, the specific surface area decreases. The specific surface area of the catalysts supported by alkaline earth metal oxides is reduced, and the catalysts supported by MgO, CaO, SrO and BaO are calcined to form oxide agglomeration, which blocks the pores of the carrier and leads to a smaller specific surface area^13,14^. The specific surface area of the catalysts supported by Group VIII metal oxides is different. Only the specific surface area of the Cr_2_O_3_/γ-Al_2_O_3_ catalyst increases to 338.43 m^2^/g. The specific surface area of CuO/γ-Al_2_O_3_, ZnO/γ-Al_2_O_3_, Mo_2_O_3_/γ-Al_2_O_3_ and Mn_2_O_3_/γ-Al_2_O_3_ decreases, and the oxides formed due to the decrease in specific surface agglomerate during the calcination process, blocking the catalyst voids^21,22^. It can be seen from Table 3 that the specific surface area of the γ-Al_2_O_3_ catalyst increases after Fe_2_O_3_, Co_2_O_3_ and Cr_2_O_3_ are loaded, and the increase in specific surface area promotes better adsorption of tar molecules into the catalyst and contact with the active site for catalytic cracking reaction to improve the catalytic efficiency of the catalyst.

**Table 3:** Specific surface area of different metal oxide supported γ-Al_2_O_3_ catalysts (m^2^/g).

Subgroup metal oxides	Specific surface area	Alkaline earth metal oxides	Specific surface area	VIII metal oxides	Specific surface area
γ-Al_2_O_3_	271.64	MgO/γ-Al_2_O_3_	203.21	CuO/γ-Al_2_O_3_	200.34
Fe_2_O_3_/γ-Al_2_O_3_	301.88	CaO/γ-Al_2_O_3_	211.25	ZnO/γ-Al_2_O_3_	198.12
Co_2_O_3_/γ-Al_2_O_3_	319.67	SrO/γ-Al_2_O_3_	235.25	Mo_2_O_3_/γ-Al_2_O_3_	247.99
Ni_2_O_3_/γ-Al_2_O_3_	250.20	BaO/γ-Al_2_O_3_	260.57	Cr_2_O_3_/γ-Al_2_O_3_	338.43
				MnO_X_/γ-Al_2_O_3_	215.77

## Conclusion

The coal sample was pyrolyzed at 450 °C, 500 °C, 550 °C and 600 °C and it is found that the maximum tar yield was 0.32 g at 600 °C. With the increase of pyrolysis temperature, the proportion of most of the light fractions in the collected coal tar shows a decreasing trend, and the proportion of washing oil and bitumen shows an increasing trend. With the increase of constant temperature pyrolysis time, the tar yield does not change much, while the pyrolysis gas yield increases significantly. The tar yield of constant temperature pyrolysis for 15 min increases by 15.63% compared with 0 min. With the increase of the constant temperature time, the asphalt component in the tar increases, mainly because the increase of the pyrolysis time is conducive to the overflow of macromolecular substances in the coal, so that the light tar content in the tar component decreases. The coal samples were mixed with γ-Al_2_O_3_ catalysts supported by different groups of metal oxides for mixed pyrolysis. The Fe_2_O_3_/γ-Al_2_O_3_ catalyst made the tar yield the highest, which was 0.75 g, which was 134.38% higher than that of raw coal pyrolysis. From the increase of light oil and phenol oil and the decrease of anthracene oil and pitch, Co_2_O_3_/γ-Al_2_O_3_, Fe_2_O_3_/γ-Al_2_O_3_ and Cr_2_O_3_/γ-Al_2_O_3_ have better effect on the quality improvement of coal mixed pyrolysis tar. The specific surface area of the catalyst has a great influence on the mixed pyrolysis product of the catalyst and coal, and the larger the specific surface area, the better the pyrolysis effect.

## Data Availability

All data generated and analysed during this study are included in this published article.

## References

[1] Hou WL, Dong YH (2022). Research on complementary development of traditional energy and new energy in Hainan Province. Intell. City..

[2] Xu XY, Dong Y (2021). Tripartite evolutionary game analysis of energy industry structural transformation. J. Anhui Univ. Technol. (Nat. Sci.).

[3] Zhang L, Chen JH, Zhang L (2017). Preparation of hydrogen-rich gas by heavy tar cracking with pyrolysis coke catalyst modified by plasma. Energy Sources Part A Recov. Util. Environ. Effects.

[4] Song WL, Du L, Lin WG (2018). Low rank coal pyrolysis poly-generation and hybrid power system. Chin. J. Process. Eng..

[5] Deng YC, Tang XL (2021). Status and development of classification utilization technology of coal pyrolysis. Coal Chem. Ind..

[6] Zhang L, Chen JH, Zhang L (2019). Preparation of Mn-CoO/ supported pyrolysis coke catalyst with plasma and its application in SCO denitration process. Pol. J. Environ. Stud..

[7] Wang XD, Han JZ, Lu JY (2012). Catalytic cracking of coal pyrolysis product for oil and gas upgrading over char-based catalysts. CIESC J..

[8] Liu JH, Hu HQ, Jin LJ (2010). Integrated coal pyrolysis with CO_2_ reforming of methane over Ni/MgO catalyst for improving tar yield. Fuel Process. Technol..

[9] Liao HQ, Wu HD, Yu J (2016). Effect of metal oxides on catalytic pyrolysis of Huolin River lignite. J. Wuhan Univ. Sci. Technol..

[10] Wang RC, Sun M, Liu QX (2011). Extraction and GC/MS analysis of phenolic compounds in low temperature coal tar Northern Shaanxi. J. China Coal Soc..

[11] Cui, L. P. & Cai, H. H. The effect of supported catalysts on the gaseous product during the pyrolysis of Huangling coal. In *The 14th Chinese Chemical Society National Conference on Fluorine Chemistry.* Vol. 211 (2016).

[12] Chen JH, Li Y, Zhang L (2022). Study on the migration of sulfur in coal coke by Fe_2_O_3_/γ-Al_2_O_3_ catalyst. Fresenius Environ. Bull..

[13] Chen JH, Li Y, Zhang L (2022). Study on Cr_2_O_3_/γ-Al_2_O_3_ catalysts for mixed pyrolysis of coal to produce hydrogen-rich fuel gas. Pol. J. Environ. Stud..

[14] Chen JH, Xia F, Li Y (2022). Study on the preparation of hydrogen-rich fuel gas from mixed pyrolysis of coal by alkaline earth metal oxide supported γ-Al_2_O_3_ catalyst. Pol. J. Environ. Stud..

[15] Zhang L, Chang X, Chen JH (2020). Preparation of NiO/PC catalyst with plasma for cracking tar to produce flammable gas. Int. J. Hydrogen Energy.

[16] Chen JH, Li Y, Chen XJ (2021). Study on calorific value of semi-coke and desulfurization effect during pyrolysis of bituminous coal. Fresenius Environ. Bull..

[17] Shi QM, Mi YC, Wang SM (2022). Trap chatacteristic and mechanism of volatiles during pyrolysis of tar-rich coal. J. China Coal Soc..

[18] Li Y, Li K, Zhang ZX (2021). Research progress on catalytic of biomass with alkaline earth metal oxide-based catalysts. Biomass Chem. Eng..

[19] Li Z, Zhao XC, Miao BB (2014). Preparation of supported Fe_2_O_3_/γ-Al_2_O_3_ catalyst and its performance in microwave pyrolysis of coal. J. Mater. Sci. Eng..

[20] Zhong LD, Wu YJ, Wang L (2022). Study of CoOx over Pd supported catalysts for CO and C_3_H_8_ oxidation. J. Shaanxi Normal Univ. (Nat. Sci. Ed.).

[21] Gao H, Dong YC, Zhou SY (2019). Thermocatalytic decomposition of a sarin simulating agent by metal oxides supported on γ-Al_2_O_3_. Chin. J. Environ. Eng..

[22] Xu RY, Yang ZN, Niu YX (2022). Removal of microplastics and attached heavy metals from secondary effluent of wastewater treatment plant using interpenetrating bipolar plate electrocoagulation. Sep. Purif. Technol..

